# Gutta Percha, as an Application to Clasps

**Published:** 1856-10

**Authors:** Edmund Osmond

**Affiliations:** Resident Dentist to the Infirmary of the Ohio College of Dental Surgery, Dental Infirmary, College St.


### GUTTA PERCHA, AS AN APPLICATION TO CLASPS.

BY EDMUND OSMOND, D. D. S.,

*Resident Dentist to the Infirmary of the Ohio College of Dental Surgery.*

One of the most formidable objections to the use of clasps for securing artificial dentures is, the galvanic action which inevitably ensues from their application, in consequence of the retention of food and viscid secretions between them and the teeth to which they are applied. An acid is produced as the result, and the destruction of the teeth follows, sooner or later, according to their density and the strength of the acid produced. A second objection is, the periostitis frequently produced and consequent absorption ; indeed, in some patients it matters not how careful the operator may be that his clasps do not strain the tooth, the mere contact of them is sufficient to produce irritation. Hence, we are debarred from adopting this (otherwise in the majority of cases), the most satisfactory method of inserting partial sets, and extract teeth which are still sound, for the purpose of adapting an atmospheric plate ; and even then the teeth thus inserted cannot, from the position of the antagonists, be inserted so as to be as useful as a "clasp job."

It detracts much from the satisfaction of both dentist and patient to know that the teeth thus inserted with clasps, now so useful, are doomed to be, in a comparatively short time, perfectly useless, and a source of disease to the remaining dentures.

I propose, in the present article, to submit to my brethren of the profession a plan I have lately pursued, and if they find, upon testing it, that it will remedy the evils above mentioned, I shall feel amply rewarded for my trouble.

Gutta Percha is, I presume, pretty well known in the profession by this time. I purpose applying it as a coating to clasps, for these reasons, viz :

1st. Its incorruptibility (if pure).

2d. It is a non-conductor of electricity.

3d. Its semi-elasticity, which entirely prevents periostitis and mechanical action.

4th. Its superiority over "tubing," being practicable where "tubing" is not—being also a firmer mode of attachment.

5th. Its perfect cleanliness, and the ease with which its presence its tolerated by the most irritable gums.

My manner of applying it is as follows :

First get up cast, and fit the plate to the necks of the teeth, in the usual manner. Then punch small holes with a fine punch around the border of the plate, fitting the necks of the teeth to which you intend attaching your clasps. A

double row of fine holes extending from two to four lines from the border is sufficient. Next, lay a small piece of lead upon the male cast. Let it be broad enough to extend a little farther than the holes you have made in the borders of the plate. Replace the plate and swage it. You will then have your plate raised from your cast around the borders of the clasp teeth. This is done to allow for the gutta percha, which will fill up the space produced. Your space may be made greater or less, according to the thickness of gutta percha you design using. Next, punch a row or two (according to the size of the clasps) in the clasp stuff, and bend them to fit, in the usual manner, only loosely, and so as not to touch the teeth. This is to allow for gutta percha. Next, fit the plate in the mouth, using a stiff cement of wax and resin. Adjust so as to leave a vacancy between the tooth and clasp, and see that there is sufficient between the plate and gum around the tooth. Remove and put in plaster, in the usual manner, and solder the clasps to the plate. Previous to soldering put little strands of asbestos in the holes you have made, for the purpose of preventing the solder from filling them up.

After your teeth are on and the job soldered, scraped, and finished, round off the edges of the plate where they join the clasps, and, where you can, bevel them from the clasps. Dissolve the gutta percha in chloroform and give a few coats with a camel hair pencil, until thick enough, previously warming the plate. Let each coat dry thoroughly before the next is applied. In applying it be careful to let it run into the holes. Both sides of the clasps and plate around the teeth are to be coated. The gutta percha in the holes is the bond of union. When the last coating is dry, warm it to give it more density, and cool in cold water.

By extending the gutta percha to the plate instead of letting it terminate on the inner edge of the clasp, and by beveling that edge, the tendency to peel off when pushing the clasp on the tooth is effectually prevented. The gutta percha may be put on as thick or as thin as desired, and may even be so applied as to render it almost impossible for substances to get between it and the tooth—although should they do so, galvanic action will not take place by reason of the clasp.

I have tested the above in this Infirmary, and in one very bad case for clasps in which absorption had taken place, to a great extent, in consequence of salivary calculus; the teeth to which I clasped being the two anterior superior molars, having two large fillings in them. I inserted nine teeth, being front teeth and bicuspids, the patient having no molars below. She wears them with great comfort, uses them entirely in mastication, and has not once complained of the slightest soreness, although when I filled her clasp teeth, the cavities being approximal, it gave her great pain.

Even should the gutta percha wear off a little in time, which thing I have not tested sufficiently to know, it can be readily replaced and with little trouble.

Since writing the above, a far better plan has suggested itself to my mind; one that cannot possibly fail. I have experimented upon it, and with success. However, as the other may be applicable where this is not, as in the cases of very narrow clasps, I give both plans. My second method and the best, is this:

Get up casts and strike up a plate to fit, in the usual way, fitting it as for ordinary clasps. Now, take a piece of plate thinner than that you use for the base, cut a strip about half as broad again as you wish your clasp to be. Next, take a fine piece of card-board, cut a strip from it a little less than the breadth of your clasp, lay this upon the strip of plate and bend the edges of the latter upon your card-board, and hammer it close with a wooden hammer. This is your clasp stuff, which you will fit to the teeth in the usual manner, having the card-board next the teeth when you have fitted them to the teeth, and plate in the mouth with stiff wax. Solder them to the plate in the usual manner. In soldering the card-board will burn out, leaving the middle of the clasp, next the teeth, hollow. A piece of clasp, viewed from the end, will present the appearance of the small Italic latter *c* flattened. When you have finished the job and polished it, in the usual manner, you will fill up the hollow space in the clasp with gutta percha, swelling it out from the edges of the clasp, so as to keep them from touching the tooth.

The best way to do this is, first, put a coat or two of gutta percha, dissolved in chloroform, in the groove, taking care to apply it well underneath the overlapping edges. After this has dried, you may fill up the groove with gutta percha, softened by heat, using a plugger to shape it to your liking. Let it cool and then apply to the mouth.

The gutta percha, when applied in this manner, forms a semi-elastic cushion to the tooth, and cannot possibly become loose, as it is retained by the edges of the clasp. Another advantage is, that the edges of the clasp are rounder and susceptible of a finer finish than it is possible to give to those of an ordinary clasp. India rubber, or anything else which may answer the purpose of preventing galvanic action and mechanical attrition, can be placed in the groove when thus constructed.

Dental Infirmary, College St., Sept. 2d, 1856.

